# Polar Organic Pollutants in Groundwater: Experimental Approaches to Biodegradation During Subsoil Passage

**DOI:** 10.1100/tsw.2002.207

**Published:** 2002-05-08

**Authors:** T.P. Knepper

**Affiliations:** ESWE-Institute for Water Research and Water Technology, D-65201 Wiesbaden, Germany

**Keywords:** polar organic micropollutants, analysis, biodegradation, laboratory scale fixed-bed bioreactor, LC-MS

## Abstract

A selection of polar organic compounds was investigated for their biodegradation on a laboratory scale fixed-bed bioreactor and the decline of the parent compounds besides the formation of metabolites was monitored. Of particular interest was the investigation into the degradation of pesticides, especially isoproturon (IPU), surfactants and industrial by-products of chemical synthesis. The results from the laboratory degradation experiments are compared to findings in groundwater.

## INTRODUCTION

Polar organic industrial chemicals emitted by wastewater discharges have been recognized for just a few years, so there is still a lack of knowledge concerning this kind of pollution[1]. The knowledge about their formation and behavior in the aquatic environment as well as degradation and toxicity is quite low[2].

The problems associated with this group of pollutants, which includes surfactants, pesticides, chelating agents, and their degradation products, is strongly related to their polarity, which enables them to bypass natural as well as manmade filtration steps. Therefore, because some of these compounds are also resistant to degradation, they are found in ground and drinking water[3,4].

Most waterworks along the river Rhine purify river water by bank filtration. The water is withdrawn out of wells. The depression cone around a well allows river water to seep into the aquifer. While the water is moving towards the wells, microbial degradation takes place and the water undergoes natural purification.

It is of crucial importance for the production of such drinking water to gain knowledge about the biodegradation of such compounds in surface water and during subsoil passage. Because the metabolite concentration can be the same order as the parent compound, these need to be analyzed simultaneously and biodegradation pathways need to be studied[5,6].

Because the concentrations of the investigated compounds — already in surface water — are in the ng/L- to low µg/L-range, and even lower in groundwater, there was a need to study their biodegradation in model systems, which allowed the analysis of these low concentrations as well as investigation of metabolism. The focus of these biodegradation assays was also to investigate adaptation processes of microorganisms involved in the breakdown, even at picomolar concentrations[7,8].

## EXPERIMENTAL METHODS

The progress of aerobic biodegradation and subsequent formation of metabolites was studied in a laboratory scale fixed-bed bioreactor (FBBR) after spiking the parent compounds at concentrations of 10 µg/L into river water taken as part of the preparatory phase (Fig. 1). After enrichment utilizing solid phase extraction, the fate of parent compounds besides the predicted formation of metabolites was pursued by reversed-phase liquid chromatography-electrospray-(tandem) mass spectrometry (LC-MS) and gas chromatography-mass spectrometry (GC-MS).

The analytical methods for the determination of the polar pollutants after several simple preparation and enrichment steps are mainly GC-MS after derivatization as well as LC-MS.

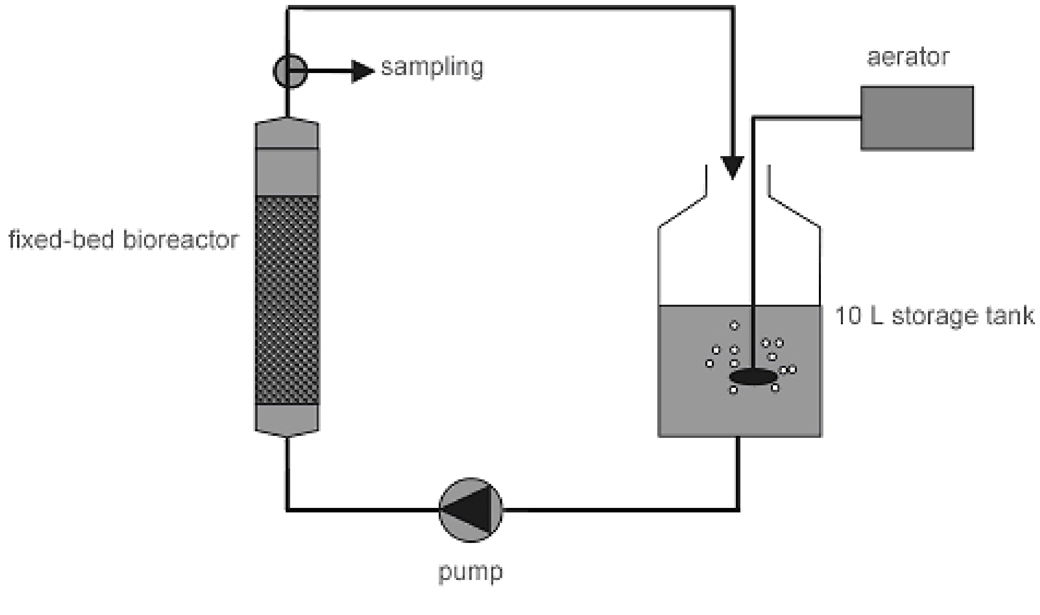

**FIGURE 1.** Scheme of laboratory scale fixed-bed bioreactor (FBBR).

For the structure elucidation of the metabolites via electrospray-MS, a series of instrument-specific MS and MS/MS registration modes were applied. For example, cone voltage and ionspray polarity were switched permanently at very short intervals within the same run. In the case of analysis performed with GC-MS, metabolite information was obtained through the aid of mass spectral databases and application of different derivatization reagents.

## RESULTS AND DISCUSSION

A selection of polar organic compounds was investigated for their biodegradation and the decline of the parent compounds besides the formation of metabolites monitored. Several degradation curves were obtained in the range between several hours and up to 30 days, whereas some compounds, such as ethylenediamine tetraacetate (EDTA) or atrazine, were confirmed as non-biodegradable under these conditions. For other compounds, such as linear alkylbenzene sulfonates, the theoretical predicted formation of several metabolites could be confirmed through the interpretation of obtained mass spectra. Thereby it was possible to verify the omega- and beta-oxidation through identification of $\alpha,\beta$-unsaturated sulfophenylcarboxylates[5]. Of particular interest was the investigation into the degradation of pesticides, such as isoproturon (IPU), for which it was found that initial concentrations less than 10 ng/L already present in the river Rhine led to an adaptation of the IPU degrading microorganisms, whereas in river water where IPU could not be detected, no degradation of the spiked IPU was observed (Fig. 2)[7].

Finally, the results from the laboratory degradation experiments were compared to findings in ground water. For example, the poorly degradable branched alkylbenzenesulfonates (ABS), which are still present in Philippine surface waters[9], could also be detected in high concentrations in groundwater samples.

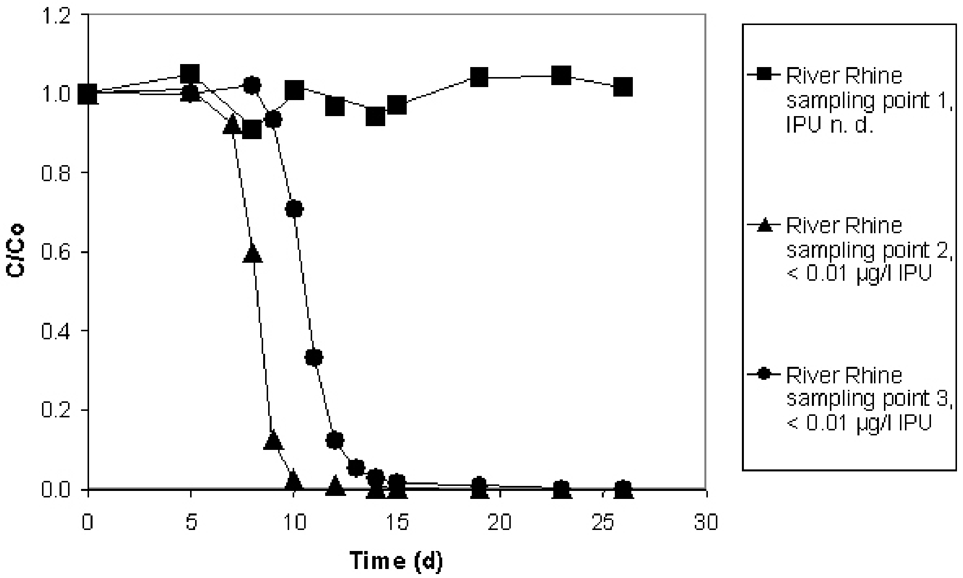

**FIGURE 2.** Follow-up of the primary degradation of IPU on three different cleaned and sterilized FBBRs prior to spiking each with 10 µg $L^{-1}$ IPU. IPU was spiked into river Rhine water taken at three different sampling points along the river Rhine. n. d. = not detected.

## CONCLUSIONS

Fixed-bed bioreactors are excellent tools to characterize polar micropollutants with regard to their biodegradation. Poorly biodegradable polar pollutants and recalcitrant degradation products being tracked in these studies can also be found in groundwater, if not adsorbed during the subsoil passage.

## ACKNOWLEDGMENTS

This paper was presented at the CSIC/ESF workshop Analysis, Toxicity and Biodegradation of Organic Pollutants in Groundwater from Contaminated Land, Landfills and Sediments, Barcelona, Spain, November 8–10, 2001.
